# In vitro dissolution and release kinetics of multisource ciprofloxacin 500 mg tablets in West Gondar, Ethiopia: Implications for interchangeability and regulatory oversight

**DOI:** 10.1371/journal.pone.0350283

**Published:** 2026-05-28

**Authors:** Tewodros Denekew Haile, Ayenew Ashenef, Chilot Abiyu Demeke, Yeniewa Kerie Anagaw, Abibo Wondie Mekonen, Minichil Chanie Worku, Asnakew Mulaw Beirhun, Yesuneh Tefera Mekasha, Melaku Getahun Feleke

**Affiliations:** 1 Department of Pharmaceutical Chemistry, School of Pharmacy, College of Medicine and Health Sciences, University of Gondar, Gondar, Ethiopia; 2 Department of Pharmaceutical Chemistry and Pharmacognosy, School of Pharmacy, College of Health Sciences, Addis Ababa University, Addis Ababa, Ethiopia; 3 Center for Innovative Drug Development and Therapeutic Trials for Africa (CDT-Africa), College of Health Sciences, Addis Ababa University, Addis Ababa, Ethiopia; 4 Department of Pharmaceutics, School of Pharmacy, College of Medicine and Health Sciences, University of Gondar, Gondar, Ethiopia; 5 Department of Veterinary Pharmacy, pharmaceutical Supply chain Management, University of Gondar, Gondar, Ethiopia; 6 Department of Veterinary Pathobiology, College of Veterinary Medicine and Animal Sciences, University of Gondar, Gondar, Ethiopia; 7 Bioequivalence Center, Clinical Trial Directorate, Armauer Hansen Research Institute, Addis Ababa, Ethiopia; 8 Department of Veterinary Pharmacy, Pharmaceutical Analysis, and Quality Assurance, University of Gondar, Gondar, Ethiopia; University of Nairobi Faculty of Health Sciences, KENYA

## Abstract

**Background:**

*In vitro* dissolution is a critical quality control test in pharmaceutical development, used to measure the rate and extent at which an active pharmaceutical ingredient (API) is released from solid dosage forms, such as tablets, into solution under standardized conditions. It serves as a predictive tool for in vivo drug behavior, providing insights into drug release mechanisms and ensuring the development of robust, effective, and consistent products. Despite its importance, data on dissolution performance remain limited for certain drugs. As a rapid, reliable, and cost-effective method to estimate drug absorption, this study focused on evaluating the dissolution profiles of different brands of ciprofloxacin 500 mg tablets and applying kinetic modeling to support regulatory decision-making.

**Methods:**

Twenty samples of ciprofloxacin 500 mg tablets were collected from retail outlets in six selected sites of the West Gondar Zone including Humera, Mai Kadra, Metema, Gendawuha, Kokit, and Midregenet towns between January and July 2022. The samples were obtained from purposively selected outlets using prescription papers issued by the University of Gondar Comprehensive Hospital, following a mystery shopper approach in line with WHO (2015) strategies. All collected samples were transported to the Ethiopian Food and Drug Authority (EFDA) laboratory and conducted as per USP specification. The amount of ciprofloxacin released was quantified using UV-Vis spectrophotometry at 276 nm. While the onset of pharmacological action was not directly measured, the Mean Dissolution Time (MDT) values suggested differences in release rates, which may influence the onset of action in vivo were considered. Dissolution profiles were evaluated using both model-independent parameters (difference factor f₁, similarity factor f₂, dissolution efficiency, and mean dissolution time) and model-dependent kinetics. Results were presented in tables, figures, and narrative form.

**Results:**

All of the included tablet brands complied with single-point dissolution study test, as their Active Pharmaceutical Ingredient release were greater than 80% within the specified 30 minutes time frame according to USP standards. Only 4 brands (21.05%) met the f1/f2 similarity criteria. However, fifteen brands (78.95%) were not interchangeable with the comparator, as the difference factors (f1) were greater than 15% and the similarity factor (f2) were less than 50%. The mean dissolution time values ranged between 1.02 and 7.16 minutes, and the results showed that four of the twenty brands (4/20) exhibited the fastest dissolution rate and onset of action. The evaluated brands followed the Korsmeyer-Peppas and Weibull curve approach (the highest coefficient of determination) for the release of drug substances from the dosage forms.

**Conclusion:**

The study reveals that while all tested ciprofloxacin 500 mg tablet brands complied with USP single-point dissolution criteria, only 21.05% met the similarity factor (f₂) and difference factor (f₁) standards for interchangeability with the comparator product. Significant variability in dissolution profiles among brands raises concerns regarding bioequivalence and potential implications for therapeutic efficacy and antimicrobial resistance. These findings underline the necessity for enhanced regulatory oversight, including routine dissolution profiling and, where appropriate, in vivo bioequivalence studies to ensure the quality and interchangeability of multisource ciprofloxacin products in Ethiopia and similar settings.

## Introduction

Antibiotics are among the most commonly prescribed drugs worldwide and remain vital for treating infectious diseases, which continue to be a major cause of illness and death, especially in low- and middle-income countries (LMICs) [1]. Ciprofloxacin, a fluoroquinolone with broad-spectrum activity against Gram-negative and certain Gram-positive bacteria, is extensively used to manage urinary tract, respiratory, and gastrointestinal infections and is included in the World Health Organization (WHO) Model List of Essential Medicines [2]. Nonetheless, the clinical effectiveness of antibiotics relies not only on their inherent pharmacological properties but also on the quality, safety, and equivalence of the formulations available on the market [3].

The circulation of substandard or non-equivalent generic medicines has become a significant public health concern, especially in LMICs where regulatory systems, local manufacturing control, and post-marketing surveillance are often weak [4,5]. The use of poor-quality antibiotics may result in subtherapeutic drug concentrations, leading to treatment failure and, more critically, fostering the emergence and spread of antimicrobial resistance (AMR) [6,7]. Since AMR already poses a serious health challenge in Ethiopia and much of sub-Saharan Africa. It is essential to ensure that ciprofloxacin tablets available on the market provide consistent quality and reliable therapeutic effectiveness [8,9].

According to the Biopharmaceutical Classification System (BCS), ciprofloxacin is classified as a Class IV drug, meaning its low solubility, and low permeable can influence bioavailability following oral administration [10]. This classification highlights that both dissolution and intestinal permeability may restrict absorption, thereby making *in vitro* dissolution testing a crucial approach for predicting *in vivo* performance [11]. For drugs in this category, establishing therapeutic equivalence among multisource formulations is particularly challenging, while conducting *in vivo* bioequivalence studies in LMICs is often constrained by financial and technical limitations [12]. As a result, in vitro approaches such as similarity (f2) and dissimilarity (f1) factors, dissolution efficiency (DE), and mean dissolution time (MDT) serve as important surrogate measures for evaluating equivalence and interchangeability [13,14]. Additionally, kinetic modeling (including zero-order, first-order, second-order, Higuchi, Hixson Crowell, Korsmeyer Peppas, and Weibull models) helps to elucidate drug release mechanisms, thereby offering deeper mechanistic understanding of dissolution profiles [15].

Ethiopia serves as a key context for this type of evaluation, as the nation depends predominantly on imported generics to satisfy healthcare needs, while its domestic pharmaceutical manufacturing remains underdeveloped [16]. Both research findings and regulatory reports have highlighted the presence of substandard medicines and inconsistencies in therapeutic quality within the Ethiopian market [17]. Post-market surveillance for selected antimicrobials conducted at the Ethiopia-Sudan-Eritrea border (around Gondar) revealed significant quality concerns. Among 71 sampled antimicrobials (16.9%) failed dissolution tests [18]. Additionally, evidence from Ethiopia indicates that none of the seven brands of amoxicillin–clavulanate potassium tablets evaluated in Hawassa town were interchangeable with the innovator product as the similarity criterion, with f₂ values below 50% [19]. Given the significant burden of infectious diseases and the growing threat of AMR to treatment effectiveness, evaluating the equivalence and interchangeability of marketed ciprofloxacin tablets is both a regulatory necessity and an urgent public health concern [20].

The relevance of this issue extends beyond Ethiopia to many other LMICs, where frequent drug shortages often necessitate brand substitution in the absence of sufficient evidence of equivalence [21]. Consequently, dissolution profiling and kinetic modeling based interchangeability studies are crucial for guiding rational procurement, maintaining continuity of care, and reducing the risks of therapeutic failure or antimicrobial resistance when substitutions occur [22].

This study investigates the *in vitro* dissolution profiles and release kinetics of multisource Ciprofloxacin 500 mg tablets marketed in the West Gondar Zone, Ethiopia. Using both model-independent-and model-dependent analytical approaches, the research evaluates the therapeutic interchangeability of 20 commercially available brands with a designated comparator product. Tablet samples were collected from retail outlets across five selected sites. All laboratory analyses were carried out at the Ethiopian Food and Drug Authority (EFDA) using pharmacopeia specifications and validated analytical methods.

In this context, the present study was undertaken to assess the in vitro dissolution profiles of ciprofloxacin 500 mg tablets available in Gondar, Ethiopia, employing similarity and dissimilarity factors, dissolution efficiency, mean dissolution time, and kinetic modeling techniques. The outcomes are expected to generate evidence that can strengthen regulatory oversight, inform procurement practices, and support the safe interchangeability of ciprofloxacin formulations in Ethiopia and comparable LMIC settings.

## Materials and methods

### Study setting, and period

The sample collection for the study was conducted in Setit Humera, West Gondar Zones, located in North-West Ethiopia, from January to July 2022. The sample sites for medicine collection include Humera, Mai Kadra, Metema, Gendawuha, Kokit, and Midregenet towns. Setit Humera is a zone bordering Sudan and Eritrea. The West Gondar Zone borders Sudan. Because of their geographical location, both Setit Humera and West Gondar have the potential for medicine smuggling and illegal border trade. Laboratory work was conducted in the Ethiopian Food and Drug Administration (EFDA) laboratory.

### Chemical, reagents and Instruments

HCl, USP ciprofloxacin RS (Lot No. R05170, Germany with a potency of 98%). Ultrasonic cleaner Ultrasonic cleaner (Dahan Scientific Co., Ltd, Korea), pH meter (Biby scientific Ltd. Co., UK), Shaker (Germany), UV–Visible spectrophotometer (Shimadzu Corporation, Japan), Erweka dissolution tester (Erweka GmbH, Germany), beakers, volumetric flasks, conical flasks, measuring cylinders, pipettes, and funnels were used in this study.

### Sample collection

Purposive sampling method was used to obtain the samples that were included in the survey. All samples were purchased from governmental and private drug retail outlets at six sites in West Gondar Zone. From all sites, a total of 20 samples of ciprofloxacin tablets were collected Prescription papers were obtained from the University of Gondar Hospital, and a mystery shopping technique was used in the collection of the samples [23].

As a result of the unavailability of the ciprofloxacin innovator product in the Ethiopian pharmaceutical market, the brand Cip001* was chosen as the comparator product. The selection of the comparator product was based on the WHO Guideline on the selection of comparator pharmaceutical product for equivalence assessment of interchangeable multisource (Generic) products [24]. This guideline recommends choosing a product that has received approval in countries associated with the International Council for Harmonization (ICH), and thus, the market leader product was selected as the comparator [24]. Furthermore, the selection of the comparator was also based on the regulatory member and the drug’s release rate. China is a regulatory member of ICH regions, and brand Cip001* is manufactured in China and it released a high amount at 5, 10, 15, 20, 30, 45, and 60 min. Hence, brand Cip001* was chosen as the comparator product due to the unavailability of the pioneering innovator product of ciprofloxacin tablet in the Ethiopian pharmaceutical market. For experimental work, all samples were transported to EFDA on the same day, and the samples were kept in their original package as well as stored at room temperature until the analysis.

### Calibration curve for ciprofloxacin dissolution test method

**Fig 1:**
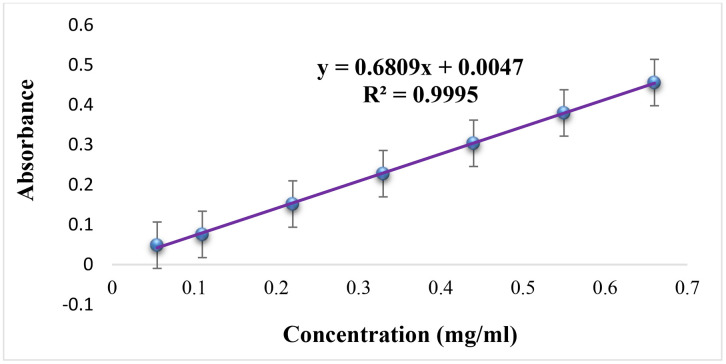
Results of Calibration curve for ciprofloxacin dissolution test method (n = 3).

A stock solution was prepared by dissolving 55 mg of ciprofloxacin USP reference standard in 50 mL of 0.01 M hydrochloric acid (HCl) to obtain a concentration of 1.1 mg/mL (USP, 2020). From this stock solution, seven concentration levels for dissolution profiling, corresponding to 10%–120% of the target concentration (0.055, 0.11, 0.22, 0.33, 0.44, 0.55, and 0.66 mg/mL), were prepared by transferring 1.25, 2.5, 5, 7.5, 10, 12.5, and 15 mL aliquots into separate 25 mL volumetric flasks and diluting to volume with 0.01 M HCl. The absorbance of each solution was measured in triplicate using a UV–visible spectrophotometer. A calibration curve was constructed by plotting absorbance against concentration of ciprofloxacin, and the resulting regression equation was used to quantify the dissolution samples (Fig 1).

### Dissolution profiles of ciprofloxacin tablets

The dissolutions of ciprofloxacin tablets were evaluated using dissolution apparatus 2 (paddle) for following the United States Pharmacopoeia protocol [25]. The dissolution medium (900 mL of 0.01 M HCl) was transferred to vessels of the dissolution apparatus. The temperature and the spindle rotation speed were set to 37 ± 0.5°C and 50 rpm, for 30 minutes. Tablets from each brand were randomly assigned to the six dissolution vessels. In accordance with FDA recommendations, a total of 12 tablets per brand were used for dissolution profiling, implying that two separate dissolution test runs (each consisting of six tablets) were conducted for each brand. Ten (10.0) mL samples were withdrawn at predetermined time points (5, 10, 15, 20, 30, 45, and 60 min). After each withdrawal, an equal volume of dissolution medium (0.01 M HCl) that had been maintained at the same temperature was replaced to maintain the total volume of the medium constant. The samples (10 mL) were immediately filtered using a filter micron size of 0.45 μm, and measured the absorbance of each sample solution was determined at 276 nm using a UV/Visible spectrophotometer. 0.01 M HCl was used as the blank solution. The amount of ciprofloxacin hydrochloride in mg (C_17_H_18_FN_3_O_3_·HCl) dissolved was calculated by the formula using Eq (1).


$$\begin{array}{c} \%\text{ Release}=\frac{AU\times CS\times D\times \text{1}\text{0}\text{0}}{AS\times L} \end{array}$$ (1)

Where;

AU = absorbance of the Sample solution

AS=absorbance of the Standard solution

CS=concentration of the Standard solution (mg/ml)

L=label claim (mg/Tablet)

D = dilution factor of the Sample solution

### Data analysis and modeling

The coded data were entered and analyzed using Microsoft Excel® 2016. Descriptive statistics were applied to summarize the results. Microsoft Excel was also used to construct the calibration curve for the reference standards and to plot the time-dependent dissolution profiles of the drug. The model-dependent parameters, including percent dissolution efficiency and mean dissolution time, were calculated using the KineDS® version 3.0 software program, while fit factors were evaluated using Microsoft Excel® 2016. The dissolution profiles of Ciprofloxacin 500 mg tablets were characterized and compared through the independent models of dissolution efficiency (DE), mean dissolution time (MDT), the difference (f1), and similarity factor (f2), as well as by the dependent models of zero- to third-order kinetics, Higuchi, Weibull, Hixon–Crowell, and Korsmeyer–Peppas as previously described [13,26–29]. In order to clarify the mechanism underlying the release of ciprofloxacin, dissolution data were analyzed using a range of kinetic models, including zero-order, first-order, second-order, third-order, Higuchi, Hixson-Crowell, Korsmeyer-Peppas, and Weibull models. The model that provided the optimal fit was determined by evaluating the highest R² and the lowest Akaike Information Criterion (AIC) values [30,31]. The best-fit model was selected based on the highest R² and lowest AIC values [30,31].

### Ethical consideration

Ethical approval was not required as no animals or human subjects were involved in this study.

## Results

### The Calibration curve for dissolution study

As revealed on the calibration curve (Fig 1), a linear regression equation was y = 69.107x − 0.0367, *r*^2^ = 0.9995. Where *Y* is the absorbance and X is the concentration in mg/mL (n = 3). The response function (calibration curve) showed a strong linear relationship between the concentration of the tested samples and the absorbance values over the concentration range of 0.055 mg/ml (10%) −0.66 mg/ml (120%).

### Dissolution test results

**Fig 2:**
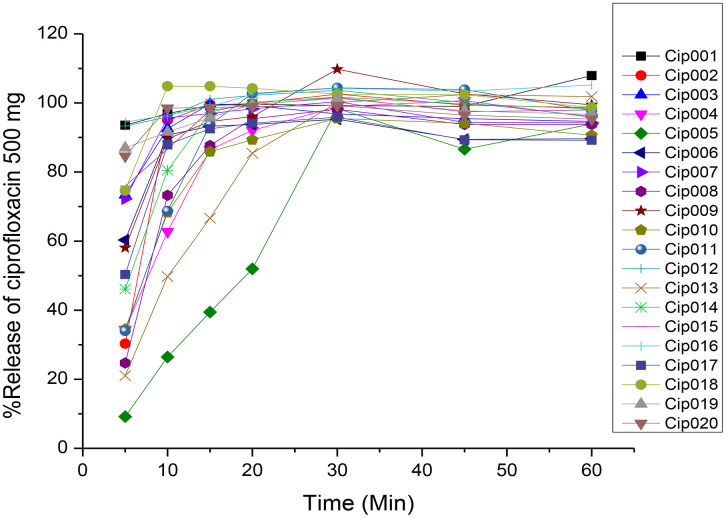
Dissolution profiles of different brands of ciprofloxacin 500 mg tablets. Cip = Ciprofloxacin 500 mg, 001–020 = the number of brands.

According to the USP 43 official monograph, ciprofloxacin should release more than 80% (Q) of the indicated amount at a single time point of 30 minutes (Table 1 and Fig 2). All 20 brands of ciprofloxacin tablets released more than 80% of their pharmaceutically active components in less than 30 minutes. These demonstrate that all test brands met the pharmacopoeia tolerance limit for single-point compliance outlined in the official United State Pharmacopoeia-43 ciprofloxacin monograph.

**Table 1:** Dissolution profile result of Ciprofloxacin 500 mg tablets evaluated in the study (n = 12).

		Sampling time (min)						
S/_NO_	Sample code	5	10	15	20	30	45	60
1	**Cip001**	93.54 ± 0.72	96.92 ± 0.63	99.28 ± 0.46	99.65 ± 0.43	99.21 ± 1.37	99.03 ± 0.82	107.92 ± 0.77
2	**Cip002**	30.31 ± 2.04	95.04 ± 0.97	99.49 ± 0.99	99.80 ± 1.01	102.58 ± 1.52	99.39 ± 0.50	98.60 ± 0.88
3	**Cip003**	73.35 ± 2.57	92.58 ± 0.62	99.71 ± 0.49	99.26 ± 0.63	96.82 ± 0.42	95.46 ± 0.53	94.66 ± 0.93
4	**Cip004**	34.43 ± 0.97	62.73 ± 0.55	86.48 ± 1.48	92.04 ± 0.93	98.91 ± 0.77	100.64 ± 0.36	95.83 ± 0.46
5	**Cip005**	9.18 ± 0.65	26.44 ± 1.29	39.43 ± 0.31	51.98 ± 0.68	98.41 ± 0.59	86.60 ± 0.76	93.91 ± 0.92
6	**Cip006**	60.31 ± 0.96	90.71 ± 0.96	93.26 ± 0.32	93.77 ± 0.40	95.30 ± 0.65	89.43 ± 1.16	89.62 ± 0.65
7	**Cip007**	72.24 ± 1.10	95.67 ± 1.15	96.94 ± 0.54	98.30 ± 0.75	100.39 ± 0.79	94.41 ± 1.04	94.27 ± 0.89
8	**Cip008**	24.71 ± 2.17	73.26 ± 0.65	87.70 ± 1.20	95.43 ± 0.68	98.18 ± 0.70	93.76 ± 0.89	93.75 ± 0.90
9	**Cip009**	58.08 ± 1.24	89.60 ± 1.42	94.64 ± 0.49	96.05 ± 0.82	109.76 ± 1.10	102.62 ± 1.36	99.47 ± 0.56
10	**Cip010**	34.51 ± 0.79	68.26 ± 1.28	85.88 ± 1.41	89.31 ± 0.28	95.50 ± 1.29	94.11 ± 0.75	90.67 ± 0.60
11	**Cip011**	34.00 ± 0.82	68.73 ± 1.11	93.70 ± 0.80	102.74 ± 1.12	104.38 ± 0.57	103.85 ± 0.84	97.81 ± 0.63
12	**Cip012**	93.33 ± 0.56	95.98 ± 1.04	101.05 ± 0.52	102.19 ± 0.56	103.67 ± 0.70	100.15 ± 0.76	96.09 ± 0.49
13	**Cip013**	21.01 ± 2.17	49.75 ± 0.76	66.62 ± 1.06	85.43 ± 0.65	100.10 ± 0.57	102.42 ± 0.70	101.86 ± 0.95
14	**Cip014**	46.09 ± 0.60	80.51 ± 0.72	97.26 ± 0.72	100.17 ± 0.87	101.71 ± 1.19	99.70 ± 0.53	98.30 ± 1.06
15	**Cip015**	75.96 ± 0.40	86.67 ± 1.28	97.87 ± 0.58	99.47 ± 1.27	101.60 ± 0.65	97.45 ± 0.90	98.00 ± 0.64
16	**Cip016**	94.44 ± 1.05	96.52 ± 0.43	98.89 ± 0.96	102.58 ± 1.19	104.28 ± 1.34	103.43 ± 0.55	105.19 ± 0.93
17	**Cip017**	50.34 ± 0.71	88.01 ± 1.08	92.50 ± 0.95	94.13 ± 0.99	95.88 ± 2.64	89.33 ± 0.78	89.20 ± 0.71
18	**Cip018**	74.61 ± 1.17	104.84 ± 1.12	104.84 ± 1.00	104.24 ± 0.69	102.53 ± 0.70	102.38 ± 0.93	98.68 ± 0.50
29	**Cip019**	86.97 ± 0.90	91.58 ± 0.84	95.74 ± 0.50	99.60 ± 1.00	101.33 ± 0.72	97.73 ± 0.82	96.68 ± 0.58
20	**Cip020**	84.61 ± 1.35	98.51 ± 0.39	98.66 ± 1.16	98.96 ± 0.84	99.25 ± 0.45	96.45 ± 0.56	95.34 ± 0.93

### Comparisons of dissolution profiles by independent model parameters

According to the FDA 1997 [27] and EMA 2010 [32] guidelines, two dissolution profiles are considered similar when f1 values are between 0–15 and f2 values range from 50–100. Based on this criterion, only four brands such as Cip012, Cip016, Cip019 and Cip020 showed acceptable similarity (f2 > 50 and f1 < 15) with the reference product and indicated that four brands of ciprofloxacin 500 mg tablets were interchangeable with the comparator product. The study revealed, only 21.05% of the brands present dissolution profiles similar to that the comparator product. Hence, only equivalent formulations can be interchangeable.

Furthermore, dissolution efficiency (DE) was applied to evaluate the drug release profiles and to determine the interchangeability of the different brands. According to the analysis, all generic brands were considered similar to the comparator product, as the difference in dissolution efficiency was within the accepted limit of less than 10% (Table 2). This finding aligns with the dissolution efficiency guideline’s acceptance threshold for therapeutic interchangeability (10%) [33]. In a pharmaceutical context, the measurement of mean dissolution time (MDT) is equally important, as it provides an estimate of the drug release rate from the dosage form and reflects the retarding effect of the polymer matrix. Among the tested products, Brand Cip004 exhibited the highest MDT (7.16), indicating the slowest drug release, while Brand Cip012 showed the lowest MDT (1.02), suggesting the fastest release (Table 2).

**Table 2:** Comparisons of dissolution profile by model independent parameters.

No.	brand	F1	F2	DE%	MDT (minute)
1	Cip001*	0	0	9.45	3.28
2	**Cip002**	22.54	21.86	8.92	5.71
3	**Cip003**	8.61	46.10	9.04	2.69
4	**Cip004**	36.62	19.83	8.44	7.16
5	**Cip005**	53.86	11.13	6.66	1.74
6	**Cip006**	15.69	35.11	8.55	2.76
7	**Cip007**	8.59	45.27	9.10	2.35
8	**Cip008**	35.92	18.55	8.30	6.87
9	**Cip009**	16.37	33.79	9.05	5.43
10	**Cip010**	28.62	23.49	8.16	5.97
11	**Cip011**	32.20	20.91	8.69	6.67
12	**Cip012**	1.01	90.71	9.45	1.02
13	**Cip013**	34.28	18.91	8.05	1.17
14	**Cip014**	22.74	26.35	8.92	5.55
15	**Cip015**	10.09	46.54	9.16	3.92
16	**Cip016**	0.58	96.55	9.50	3.01
17	**Cip017**	20.33	29.43	8.44	3.19
18	**Cip018**	11.19	45.48	9.36	3.12
19	**Cip019**	5.33	63.41	9.26	2.50
20	**Cip020**	3.84	63.61	9.26	1.75

### Dissolution profile comparison by Model dependent parameters

The study found that, the Korsemeyer-Peppas and Weibull models provided the best fit for most brands. Particularly, Cip005 and Cip013 showed high R² values under the Korsmeyer-Peppas model (0.911 and 0.844, respectively), suggesting a combined diffusion and erosion-controlled release mechanism. Cip020 demonstrated the highest R² under the third-order model (0.810), but with relatively poor AIC performance, indicating potential over fitting (Table 3).

**Table 3:** The model dependent dissolution kinetics result of tested Ciprofloxacin tablets.

Model dependent parameters		Ciprofloxacin 500 mg tablet code																			
Parameters		Cip0 01*	Cip0 02	Cip0 03	Cip0 04	Cip0 05	Cip0 06	Cip0 07	Cip0 08	Cip0 09	Cip0 10	Cip0 11	Cip0 12	Cip0 13	Cip0 14	Cip0 15	Cip0 16	Cip0 17	Cip0 18	Cip0 19	Cip0 20
**Zero order**	R^2^	0.424	0.242	0.154	0.510	0.764	0.147	0.136	0.365	0.400	0.429	0.412	0.557	0.670	0.361	0.361	0.507	0.183	0.192	0.316	0.588
	AIC	2.43	6.02	4.62	5.42	5.64	5.04	4.69	5.91	5.10	5.58	5.80	3.03	5.67	5.54	4.42	2.28	5.38	4.66	3.55	3.92
**First-order**	R^2^	0.421	0.234	0.165	0.447	0.652	0.160	0.146	0.315	0.364	0.379	0.370	0.576	0.543	3.34	0.360	0.504	0.194	0.196	0.321	0.659
	AIC	2.44	6.15	4.64	5.82	6.42	5.06	4.71	6.11	5.15	5.74	5.96	3.03	6.11	5.63	4.44	2.29	5.42	4.67	3.56	3.93
**Second -order**	R^2^	0.418	0.230	0.174	0.382	0.425	0.172	0.156	0.273	0.333	0.330	0.326	0.595	0.404	0.310	0.357	0.502	0.203	0.200	0.326	0.732
	AIC	2.44	6.69	4.66	6.37	8.13	5.12	4.73	6.96	5.27	6.17	6.47	3.03	7.99	5.87	4.48	2.29	5.53	4.70	3.57	3.93
**Third -order**	R^2^	0.414	0.23	0.183	0.326	0.293	0.182	0.165	0.247	0.307	0.290	0.288	0.616	0.310	0.284	0.354	0.499	0.209	0.203	0.330	**0.810**
	AIC	2.45	7.22	4.70	7.00	7.45	5.21	4.77	7.18	5.51	6.93	7.08	3.04	7.20	6.51	4.54	2.30	5.76	4.75	3.59	3.94
Korsmeyer-Peppas model	R^2^	0.714	0.51	0.443	0.766	**0.911**	0.430	0.415	0.623	0.667	0.699	0.690	0.288	**0.844**	0.649	0.678	0.804	0.477	0.462	0.641	0.288
	AIC	1.5	6.02	4.39	5.42	5.86	4.85	4.48	5.91	4.77	5.40	5.66	2.84	5.64	5.31	4.02	1.65	5.20	4.43	3.14	3.75
Weibull-model	R^2^	0.522	0.446	0.194	0.676	**0.878**	**0.828**	0.152	**0.868**	0.602	0.770	0.514	0.140	0.902	0.350	0.274	0.769	0.441	0.102	0.171	0.133
	AIC	1.51	5.40	4.43	3.14	4.79	5.13	4.44	5.72	3.00	4.63	4.96	3.37	4.85	5.13	3.90	2.58	4.96	4.93	3.31	3.81
Hixson-Crowell- model	R^2^	0.43	0.237	0.161	0.535	0.711	0.156	0.143	0.331	0.375	0.396	0.385	0.569	0.590	0.343	0.360	0.505	0.190	0.195	0.320	0.635
	AIC	2.63	6.08	4.83	3.69	6.25	5.06	4.70	6.01	5.13	5.67	5.88	3.03	5.91	5.59	4.43	2.28	5.40	4.67	3.56	3.92
Higuchi mode	R^2^	0.339	0.303	0.162	0.320	0.649	0.688	0.150	0.218	0.320	0.147	0.126	0.241	0.794	0.923	0.149	0.364	0.300	0.153	0.709	0.617
	AIC	6.90	6.40	6.73	5.85	5.91	6.60	6.74	6.05	6.45	5.99	6.07	6.92	5.33	6.31	6.67	6.90	6.49	6.76	8.81	6.86

Among all models, the Weibull model provided good fitting for Cip005 (R² = 0.878) and Cip008 (R² = 0.868), with moderate AIC values, suggesting this model’s flexibility in capturing various release kinetics. Meanwhile, the Higuchi and Hixson-Crowell models generally yielded lower R² values across brands, indicating a limited applicability in describing the drug release behavior for these ciprofloxacin tablets

## Discussion

In the current study, the dissolution profiles of ciprofloxacin 500 mg tablet marketed in and around Gondar, Ethiopia, were compared using a model-independent approach with parameters of fit factors (f1, f2), dissolution efficiency (DE), and mean dissolution time (MDT) and model-dependent kinetic modeling. The results are valuable for revealing the interchangeability and equivalence of multisource ciprofloxacin 500 mg products in a context where therapeutic consistency and quality assurance are critical to safeguarding treatment outcomes. According to the results of the investigation, all brands passed a single-point dissolution test and adhered to the United States Pharmacopeia (USP) criteria by releasing over 80% of the formulation in less than 30 minutes.

The factors of similarity (f2) and dissimilarity (f1) are extensively recognized regulatory tools for the comparative analysis of dissolution profiles. According to the US FDA and EMA guidelines, f2 values between 50 and 100 indicate similarity, while f1 values below 15 suggest no significant difference in release profiles [27]{Moore, 1996 #44;USFADA, 1997 #15;FDA, 1997 #10}. In the present study, four brands exhibited an acceptable degree of similarity with the reference product, thereby suggesting their potential interchangeability; conversely, fifteen brands (78.95%) exhibited notable dissimilarity, which raises concerns regarding their therapeutic equivalence. Comparable findings from studies conducted in Lebanon [34], Nigeria [35] and Bangladesh [36] indicated that 80%, 50% and 36.4% of the tested ciprofloxacin 500 mg tablets were not interchangeable with the comparator products, respectively. Similarly, this finding is consistent with a previous study conducted in the United Arab Emirates, where the tested ciprofloxacin products also showed variability in interchangeability during clinical practice, as their values did not meet the f₁ and f₂ specifications [37].

Such variability among marketed products underscores the need for continued post-marketing surveillance, as the use of dissimilar products in clinical practice may result in suboptimal exposure and increase the risk of antimicrobial resistance (AMR).

The variation in drug dissolution depends on many factors, such as excipient type and quantity, dosage form manufacturing process (milling, mixing, and compression force), size of drug particles, and storage conditions [38]. The manufacturer can significantly influence the dissolution profile of a drug, even when the dosage strength and active pharmaceutical ingredient (API) are the same. This is particularly important for Biopharmaceutics Classification System (BCS) Class IV drugs like ciprofloxacin, which have low solubility and high permeability. In such cases, dissolution is the rate-limiting step for absorption, making formulation differences highly impactful [34].

For instance, a study compared 19 distinct generic formulations of simvastatin, including both tablets and capsules sourced from international manufacturers, in comparison to the United States innovator product with respect to pharmaceutical quality. The findings indicated that the manufacturing standards for the international generics were not comparable in terms of quality parameters when compared with the US innovator drug, and a notable variability was also observed among the foreign-produced tablets themselves [39].

In a similar vein, an additional investigation conducted a comparative analysis of the dissolution behavior exhibited by six distinct brands of diclofenac sodium extended-release tablets sourced from the national marketplace. The findings indicated that the release characteristics demonstrate significant variability among the various manufacturers, and even formulations that are ostensibly identical presented markedly different release profiles. Consequently, the interchangeability of these pharmaceutical preparations is questioned [40].

Another possible reason for the variation in dissolution rate pertains to the disparity in particle size or surface area of the pharmaceutical particles [41]. Solid dosage forms may exhibit either disintegration or retention of integrity upon interaction with gastrointestinal fluids subsequent to oral administration, contingent upon their design. Given that disintegration significantly influences the interface area between the solid and the liquid, it typically assumes a crucial role in the dissolution mechanism. Nevertheless, it is imperative to concede that a direct correlation between disintegration and dissolution does not inherently exist, especially in the context of poorly soluble pharmaceuticals [42].

The storage of a pharmaceutical compound may significantly influence its dissolution characteristics. For instance, a research investigation compared the dissolution rates between the branded version and its generic equivalent of diclofenac sodium after being subjected to conditions of 40°C and 75% relative humidity. The findings indicated that the dissolution rate of the generic formulation of diclofenac sodium experienced a substantial decline during the storage period in comparison to its branded equivalent [43].

Furthermore, another study conducted in Egypt identified considerable discrepancies in the in-vitro performance of omeprazole capsules. It was determined that the branded formulation exhibited greater resilience to alterations induced by the packaging materials compared to its generic counterparts [44]. The disparity in dissolution rates between branded pharmaceuticals and their generic equivalents may be ascribed to the distinct compositions of excipients utilized. This variation can predominantly affect the side effect profiles associated with the generic formulations [45]. Excipients are defined as substances that are not pharmacologically active, encompassing a range of components such as binders, fillers, disintegrants, lubricants, sweeteners, preservatives, flavoring agents, colorants, and printing inks, among others [46, 47].

Although excipients are classified as inactive components devoid of therapeutic efficacy, numerous investigations have illustrated that excipients may elicit a variety of adverse effects [46]. In numerous instances, the efficacy of a pharmaceutical product can be significantly influenced by the quality of excipients utilized in the manufacturing process as well as the overall quality of the production methodology [40]. In the scholarly literature, for instance, it has been documented that the excipients present in a specific generic formulation of simvastatin facilitated the expedited release of the active pharmaceutical ingredient during the initial five minutes of the dissolution assay [48]. Furthermore, empirical research has demonstrated that varying formulations of digoxin (a cardiac glycoside characterized by a narrow therapeutic index) produced substantial disparities in dissolution profiles. The study indicates that either batch-to-batch or amongst brands bio-in-equivalence originates from differences in dissolution rates [49]. It is widely recognized that digoxin represents a medication with a narrow therapeutic index, implying that minor fluctuations in dissolution rates could produce significant complications due to excessive adverse effects or insufficient therapeutic efficacy [49].

To provide the corresponding quantitative insights, in addition to f1 and f2, the dissolution efficiency (DE) and the mean dissolution time (MDT) were used. DE reflects the area under the dissolution curve relative to the total area if complete dissolution were achieved over the same time [26]. If the disparity in dissolution efficiency between the reference and test products is within permissible thresholds, they are deemed equivalent [27]. In the present study, all generic brands exhibited comparable characteristics to the reference products, given that the difference in dissolution efficiency is less than 10% [50].

The mean dissolution time (MDT) was considered to assess the dissolution rate of the drug and its onset of action. It serves as a parameter to characterize the drug release rate from the dosage form and to evaluate the polymer’s retarding performance [51]. The MDT values were obtained from the cumulative dissolution curves of the active pharmaceutical ingredient (API) plotted as a function of time. The findings revealed that brand Cip004 exhibited the highest MDT (7.16), while brand Cip012 showed the lowest MDT (1.02) among the evaluated products. This indicates that Cip004 demonstrates prolonged drug release with a delayed onset of action, whereas Cip012 dissolves more rapidly, resulting in a faster onset of action [52]. Generally, a higher MDT value reflects a greater ability of the polymer to retain the drug, while a lower value suggests a weaker retarding effect [53].

Furthermore, the dissolution profiles of evaluated samples were subjected to model-dependent approaches to estimate the kinetics of drug release from the dosage form. Kinetic modeling provided additional explanation regarding the mechanisms that regulate drug release from the tablets. The most suitable models exhibited variability in different products, with certain models conforming to first-order kinetics (which denotes concentration-dependent release), whereas others adhered to the Higuchi or Weibull models, indicating diffusion-controlled or complex release mechanisms, as well as Korsmeyer-Peppas when multiple release mechanisms may be involved [15,54]. The observed discrepancies in release kinetics among products are likely indicative of formulation and manufacturing variances, encompassing excipient composition, compression force, and particle size distribution [55]. Notably, the variability in release kinetics possesses clinical significance, as it may manifest as differences in the onset, extent, and duration of therapeutic efficacy.

In the present study, the model that best described the release kinetics was selected based on the highest coefficient of correlation (R²) [56]. Accordingly, the Korsmeyer–Peppas and Weibull models provided the best fit for the release of the active substances. A similar finding was reported in Bangladesh, where the Weibull model was identified as the most suitable for describing the release of ciprofloxacin dosage forms [36].

The integrative application of fit factors, DE, MDT, and kinetic modeling provide a comprehensive assessment of the equivalence in dissolution profiles. Although certain ciprofloxacin brands in the Gondar market demonstrated acceptable similarity and interchangeability the observed variability across others raises concern, especially in a setting where drug shortages frequently necessitate substitution between available products. In low- and middle-income countries (LMICs) like Ethiopia, where the surveillance of post-marketing quality is constrained and the threat of antimicrobial resistance is escalating, studies evaluating interchangeability of this kind are of paramount importance. Such investigations not only inform procurement and regulatory frameworks but also enhance the rational utilization of medicines and the preservation of therapeutic efficacy.

This study provides novel and locally relevant insights into the dissolution behavior of multisource ciprofloxacin tablets in Ethiopia, addressing a significant gap in post-market surveillance within resource-limited settings. By evaluating 20 commercially available brands using both model-independent (f1, f2, dissolution efficiency, and mean dissolution time) and model-dependent kinetics approaches, the research offers empirical evidence on therapeutic interchangeability. This is a critical concern in regions where drug shortages and cost often necessitate brand substitution.

The findings reveal that while all brands met USP single-point dissolution criteria, only about 31.6% demonstrated similarity to the comparator product. This indicating considerable discrepancy that could compromise therapeutic efficacy and contribute to antimicrobial resistance. For regulatory authorities and healthcare systems in low-and middle-income countries, this work underlines the value of affordable, in vitro dissolution profiling as a pragmatic tool for quality assurance, enabling evidence-based procurement, informed substitution practices, and strengthened oversight without the need for costly *in vivo* studies. Moreover, the study provided a transferable framework for enhancing medicine quality surveillance, safeguarding patient outcomes, and supporting antimicrobial stewardship in settings where regulatory and economic constraints pose ongoing challenges to equitable access to effective antibiotics.

### Limitations of the study

In vitro results may not predict clinical outcomes due to physiological variability, and *no IVIVC* analysis was done.

## Conclusions

All tested brands met the USP criteria for the dissolution test. Because many brands were not equivalent, this study demonstrates that a single point test may not be adequate to predict the *in vivo* performance; thus, the multiple time points seem to be a more realistic approach for post-market surveillance, since it can be a discriminatory method to predict the *in vivo* performance and to demonstrate if different formulations can be interchangeable.
