# Correction: In vitro dissolution and release kinetics of multisource ciprofloxacin 500 mg tablets in West Gondar, Ethiopia: Implications for interchangeability and regulatory oversight

**DOI:** 10.1371/journal.pone.0352706

**Published:** 2026-06-26

**Authors:** Tewodros Denekew Haile, Ayenew Ashenef, Chilot Abiyu Demeke, Yeniewa Kerie Anagaw, Abibo Wondie Mekonen, Minichil Chanie Worku, Asnakew Mulaw Berihun, Yesuneh Tefera Mekasha, Melaku Getahun Feleke

The seventh author’s name is spelled incorrectly. The correct name is: Asnakew Mulaw Berihun.
